# Identification of immune-associated biomarker for predicting lung adenocarcinoma: bioinformatics analysis and experiment verification of PTK6

**DOI:** 10.1007/s12672-024-00939-9

**Published:** 2024-04-04

**Authors:** Ren-Hui Xiong, Shuo-Qi Yang, Ji-Wei Li, Xun-kai Shen, Lu-Ming Jin, Chao-Yang Chen, Yu-Ting Yue, Zhi-Chen Yu, Qing-Yu Sun, Wen Jiang, Ming-Zheng Jiang, Xiao-Yan Wang, Shi-Xu Song, Dai Cao, Hong-li Ye, Li-Ran Zhao, Li-Peng Huang, Liang Bu

**Affiliations:** 1 School of Medicine, Xiamen University, Xiamen, 361000 Fujian China; 2 Department of Thoracic Surgery, Xiang’an Hospital of Xiamen University, School of Medicine, Xiamen University, Xiamen, 361000 Fujian China; 3 Department of Thoracic Surgery, The First People’s Hospital of Yunnan Province, Kunming, 650032 China

**Keywords:** Lung adenocarcinoma, Pan-cancer analysis, Protein tyrosine kinase 6, Experimental validation, Bioinformatics, Prognostic biomarker, Immune infiltration

## Abstract

**Background:**

Abnormal expression of protein tyrosine kinase 6 (PTK6) has been proven to be involved in the development of gynecological tumors. However, its immune-related carcinogenic mechanism in other tumors remains unclear.

**Objective:**

The aim of this study was to identify PTK6 as a novel prognostic biomarker in pan-cancer, especially in lung adenocarcinoma (LUAD), which is correlated with immune infiltration, and to clarify its clinicopathological and prognostic significance.

**Methods:**

The prognostic value and immune relevance of PTK6 were investigated by using bio-informatics in this study. PTK6 expression was validated in vitro experiments (lung cancer cell lines PC9, NCI-H1975, and HCC827; human normal lung epithelial cells BEAS-2B). Western blot (WB) revealed the PTK6 protein expression in lung cancer cell lines. PTK6 expression was inhibited by Tilfrinib. Colony formation and the Cell Counting Kit-8 (CCK-8) assay were used to detect cell proliferation. The wound healing and trans-well were performed to analyze the cell migration capacity. Then flow cytometry was conducted to evaluate the cell apoptosis. Eventually, the relationship between PTK6 and immune checkpoints was examined. WB was used to estimate the PD-L1 expression at different Tilfrinib doses.

**Results:**

PTK6 was an independent predictive factor for LUAD and was substantially expressed in LUAD. Pathological stage was significantly correlated with increased PTK6 expression. In accordance with survival analysis, poor survival rate in LUAD was associated with a high expression level of PTK6. Functional enrichment of the cell cycle and TGF-β signaling pathway was demonstrated by KEGG and GSEA analysis. Moreover, PTK6 expression considerably associated with immune infiltration in LUAD, as determined by immune analysis. Thus, the result of vitro experiments indicated that cell proliferation and migration were inhibited by the elimination of PTK6. Additionally, PTK6 suppression induced cell apoptosis. Obviously, PD-L1 protein expression level up-regulated while PTK6 was suppressed.

**Conclusion:**

PTK6 has predictive value for LUAD prognosis, and could up regulated PD-L1.

**Supplementary Information:**

The online version contains supplementary material available at 10.1007/s12672-024-00939-9.

## Introduction

Lung cancer is the leading cause of cancer mortality worldwide, approximately 85% of patients has been diagnosed as non-small cell lung cancer [1]. In addition, LUAD is the most common histologic type of non-small cell lung cancer which exhibits molecular heterogeneity, and comprehending its molecular pathways is essential for potential treatments [2]. Even if surgical treatment and diagnostic methods for LUAD have been substantially enhanced, more study will be required to understand the underlying biological mechanism and then find a strategy to circumvent it [3].

Epidermal growth factor receptor (EGFR) mutations were discovered in patients with LUAD since the first trials targeting EGFR, targeted therapy has transformed the management of lung cancer [4, 5]. Now, inhibitors of EGFR, anaplastic lymphoma kinase (ALK) and Kirsten rat sarcoma viral oncogene homolog (KRAS) has been approved for the treatment of LUAD [6]. Additionally, it is noteworthy that immunotherapy is presently regarded as the most auspicious clinical intervention for LUAD, as it has received approval for first-line treatment and has demonstrated significant enhancements in the survival rates of patients [7]. Besides, the therapy possibilities for LUAD have also been drastically altered by the recent discoveries of immune-checkpoint inhibitors (ICIs) that target programmed cell death 1 (PD-1) and programmed cell death 1 ligand 1 (PD-L1) [8]. The PD-L1 enables cancer cells escape from the immune system given that it exists on the surface of cancer cells, which can combine with PD-1 which is present on the surface of antigen-stimulated T cells [9]. Nevertheless, its clinical efficacy remains limited, and not all patients derive benefits from it. Consequently, the pursuit of optimal strategies to augment immunotherapy has become a prominent area of research [10].

Protein tyrosine kinase 6 (PTK6) commonly referred to as Breast tumor kinase (BRK), is an intracellular tyrosine kinase [11]. Similar to other SRC family kinases, PTK6 is composed of a SRC homology 3 (SH3) domain, a SRC homology 2 (SH2) domain and a tyrosine kinase domain [12]. Increased expression has been identified in estrogen receptor (ER) positive and triple negative breast cancer [13, 14]. PTK6 has been most widely studied in a variety of epithelial tumors, several studies indicate that knockdown PTK6 contribute to suppress breast cancer cell migration, invasion and metastasis [15, 16]. Activation of PTK6 could mediate the PTEN loss to promote invasive prostate cancer [17]. Multiple compounds with inhibitory effects on PTK6 have been investigated currently. The inhibitor XMU-MP-2 could suppress tumor growth in breast cancer by targeting BRK [18]. PTK6 has been studied in various cancers, however, the roles of PTK6 on LUAD progression remain controversial.

In this study, we focus on the level of PTK6 expression and its relationship with immunity in pan-cancer, especially in LUAD. We aim to discover an association between immune infiltration and PTK6. The results demonstrated that when PTK6 suppressed, PD-L1 is up-regulated. This finding indicates that combine targeted therapy with immunotherapy to achieve better therapeutic effect.

## Materials and methods

### Data collection

The mRNA expression and clinical data were obtained from The Cancer Genome Atlas (TCGA) and Pan-Cancer Atlas Hub of UCSC Xena database (https://www.cancer.gov/ccg/research/genomesequencing/tcga/) (https://xenabrowser.net), the missing and uncertain clinical data were deleted Genotype-Tissue Expression (GTEx) data were acquired from the Xena database as well. Moreover, we obtained 515 LUAD patient samples and 59 normal control samples from the TCGA database for a follow-up study. The measurement data are displayed as the mean ± SD. Unpaired t-test was used for analyzing statistical assessments. The association between PTK6 and clinical characteristic variables was analyzed using Pearson chi-squared test or Fisher’s exact test.

### mRNA, protein expression level and clinical features

‘limma’ and ‘affy’ packages in the R language were used in a sequence of preparation processes to get the data available for analysis. These procedures comprised data normalization, probe ID annotation, background correction, and missing value imputation. The ‘ggplot2’ R package was employed to visualize the data. PTK6 expression levels in pan-cancer were analyzed comparative using the Wilcoxon rank-sum test. Furthermore, Using PTK6 retrieved from the Clinical Proteomic Tumor Analysis Consortium (CPTAC) dataset, protein levels were conducted by using the University of Alabama at Birmingham Cancer Data Analysis Portal (UALCAN) database [19, 20]. The String database was employed to construct a protein–protein interaction network (PPI) for PTK6 (https://string-db.org/). Thus, the “GEPIA 2” database (http://gepia2.cancer-pku.cn/#index) was used to evaluated the relationship between PTK6 expression and tumor stage [21]. Additionally, home-for-researchers (https://www.aclbi.com/static/index.html#/) was employed to analyze the expression of PTK6 at different stages and its relationship with prognosis.

### Promoter methylation levels

The DNA methylation interactive visualization database (DNMVID) (http://119.3.41.228/dnmivd/index/) database was used to explore the relationship between promoter methylation levels and PTK6 expression [22].

### Survival analysis

The prognostic value of PTK6 were conducted by the “SangerBox 3.0” software (http://sangerbox.com/home.html) [23]. A cox proportional hazards regression model was set up using the ‘survival’ R package to analyze the relationship between PTK6 expression and prognosis in every tumor. Log-rank test was performed for statistical test to obtain prognostic significance. The survival outcomes included OS, PFI, and DSS. P-values and hazard ratio (HR) with 95% confidence intervals (CI) were determined for each cancer type.

### Functional enrichment analysis in LUAD patients

The RNA-seq data and corresponding clinical information for LUAD were obtained from the TCGA database through “TCGAbiolinks” R package. All patients were separated into two groups according to the median value of PTK6 mRNA expression data. Differentially expressed gene (DEG) analysis was performed using the “limma” package, and subsequently DEG were used for KEGG enrichment. The log2 fold change and p-value calculated by “limma” R package were used as ranking metric. The GO terms (C5 collection in GSEA) were divided into three sub-collections: biological process (BP), molecular function (MF), and cellular component (CC). It is one of the most frequently used databases for path-way annotation. The two enrichment analyses were based on the BP sub-collection, which contains 8314 genes. As for GSEA, there is no need for the screening of differentially expressed genes. For the Kyoto Encyclopedia of Genes and Genomes (KEGG) and Gene Ontology (GO) analysis, we used “clusterProfiler” R package to analyze the function of differentially expressed genes (DEGs) with p-value < 0.01 and GO term network connectivity score equal to 0.6. “ggplot2” R package was used to visualize. For GSEA, we made use of the “clusterprofiler” R package in R studio, and the C5 collection was the gene set used in the present analysis.

### Immune infiltration analysis

TIMER, xCell, MCP-counter, CIBERSORT, and EPIC represent the six most recent algorithms that have been integrated into the R software package “Immuneeconv” to evaluate the immunology score evaluation’s reliability [24–28]. The transcripts associated with the immune checkpoint are SIGLEC15, IDO1, CD274, HAVCR2, PDCD1, CTLA4, LAG3, and PDCD1LG2. extracting the expression of eight genes and calculating the immune-checkpoint-related genes’ PTK6 expression value. The home-for-researchers website applied this section. An integrated repository portal for tumor-immune system interactions (TISIDB) (http://cis.hku.hk/TISIDB/) database used to explore PTK6 expression in different immune subtypes and immune-modulators [29].

### Immune checkpoint blockade (ICB) therapy response prediction

The TIDE score is currently the most promising marker of ICB response and has been reported to have higher accuracy than PD-L1 expression levels and TMB in predicting survival outcomes in cancer patients treated with ICB drugs [29]. Subsequently, Potential ICB response of PTK6 was predicted using the Tumour Immune Dysfunction and Exclusion (TIDE) database (http://tide.dfci.harvard.edu/) and the TIDE algorithm was used to predict potential immunotherapeutic responses [30, 31]. TIDE applies a set of gene expression markers to estimate two distinct mechanisms of tumor immune evasion, including the dysfunction of tumor infiltration cytotoxic T lymphocytes (CTL) and exclusion of CTL by immunosuppressive factors. Patients with high TIDE scores exhibit a higher chance of tumor immune escape and thereby exhibit a lower response rate to ICB treatment.

### Cell culture

Human lung adenocarcinoma cell lines PC9, H1975, HCC827 and normal human lung epithelial cell line BEAS-2B were obtained from American Type Cultural Collection (ATCC). All the cell lines have been authenticated within the last three years and were mycoplasma-free. PC9 were maintained in DMEM (Procell Life Science & Technology Co., Ltd) supplemented with 10% fetal bovine serum (FBS) and 1% penicillin and streptomycin solution. H1975 and HCC827 were maintained in RPMI-1640 (Procell Life Science & Technology Co., Ltd) supplemented with 10% fetal bovine serum (FBS) and 1% penicillin and streptomycin solution. BEAS-2B were maintained in Bronchial Epithelial Cell Medium (Zhong Qiao Xin Zhou Biotechnology Co., Ltd, Shanghai, China) supplemented with 1% Bronchial Epithelial Cell Growth Supplement and 1% penicillin and streptomycin solution. These cells were cultured in an incubator with a 5% CO_2_ humidified atmosphere at 37 °C.

### Quantitative real-time polymerase chain reaction analysis

Total RNA was extracted from the cells using SteadyPure RNA Extraction Kit (Accurate Biotechnology Co., Ltd.) according to the manufacturer’s instructions. The cDNA synthesis was performed using 5X Evo M-MLV RT Master Mix (Accurate Biology, AG11706). RT-PCR experiments were conducted using 2X SYBR Green Pro Taq HS Premix (Accurate Biology, AG11701). Using Beta-actin as a control. The relative expression of the target genes relative to the control was calculated according to the 2−ΔΔCT formula. Each experiment was performed in triplicate.

### Western blotting analysis

Proteins were extracted from the cell line using Radio Immunoprecipitation Assay (RIPA) buffer (Beyotime Institute of Biotechnology) lysis buffer. The samples were centrifuged at 13000×*g* for 10 min. at 4 °C, and the supernatants were collected. Proteins were separated using 12.5% SDS-PAGE. After electrophoresis, proteins were transferred onto polyvinylidene fluoride membranes (Millipore). The membranes were blocked with blocking solution (Beyotime Institute of Biotechnology) for 1 h at room temperature. Next, the membranes were incubated using the following primary antibodies overnight at 4 °C: rabbit anti-PTK6 (1:1000, Abcam), rabbit anti-PD-L1 (1:1000; Abclonal) and mouse anti-Alpha tubulin (1:1000, Proteintech). After three washes, the membranes were incubated with HRP Goat Anti-Rabbit IgG (H+L) (1:10,000, Abclonal) for 1 h at room temperature. The blots were then visualized using enhanced chemiluminescent reagent (ECL) (LABLEAD).

### Cell counting kit-8 (CCK-8) assay

Tilfrinib was purchased from MCE. The CCK-8 kit (Lablead) was used to measure half maximal inhibitory concentration (IC50) of Tilfrinib. PC9 and H1975 cell from each group were seeded onto 96-well plates at a density of 10,000 cells/well and cultured overnight. After the indicated treatments, the cells were washed twice with PBS. Then, cells were incubated with the CCK-8 reagent at 37 °C for 2 h following the manufacturer’s procedures. An optical density at 450 nm indicated cell viability and was measured by amicroplate reader.

### Cell invasion assay

Cell invasion assays were performed with Matrigel invasion chambers (Corning). After 24 h of transfection, PC9 and H1975 cells were harvested, resuspended in 200 µL serum-free medium, and seeded onto the upper chamber at density of (100,000/well). Then, Tilfrinib (0 μM, 10 μM, 20 μM) was used to treat the cells for 24 h. Cell culture medium supplemented with 10% FBS was added to the lower chamber to stimulate cell invasion. After 24 h of incubation, invaded cells were fixed with 4% paraformaldehyde. The invaded cells were stained with crystal violet (0.1%) for 15 min. Images were captured under an Olympus microscope, and the invaded cell were counted from three microscopic fields.

### Wound healing assay

After 24 h of transfection, PC9 and H1975 cells were seeded onto 6-well plates and cultured until the cells reached 90% confluence. A sterile 100 μL pipette tip was used to scrape the wound. The cells were treated with culture medium supplemented with Tilfrinib (0 μM, 10 μM, 20 μM). After 24 h of incubation, the cells were fixed with 4% paraformaldehyde. The migration rate was measured based on the migration distances.

### Apoptosis assay

Apoptotic cells were determined by flow cytometry (Beckman CytoFlex S) after the cells were doubled stained with Annexin V and propidium iodide (PI). The Annexin V-FITC Apoptosis Detection Kit (Meilunbio) was used for the apoptosis assay as per the manufacturer’s protocols.

## Statistical analysis

Statistical analysis was performed using SPSS (version 23.0, Chicago, IL), GraphPad Prism (version 8, San Diego, CA) and R (v4.0.3) software. Continuous variables were expressed as mean ± standard deviation (SD), and differences between the two groups were compared using Student’s t-test for normally distributed variables. A Kruskal–Wallis H test with Dunn’s multiple-comparisons test was used for continuous variables with a non-normal distribution, and the results were presented as the median and interquartile range.

## Results

### PTK6 expression in LUAD

**Fig. 1:**
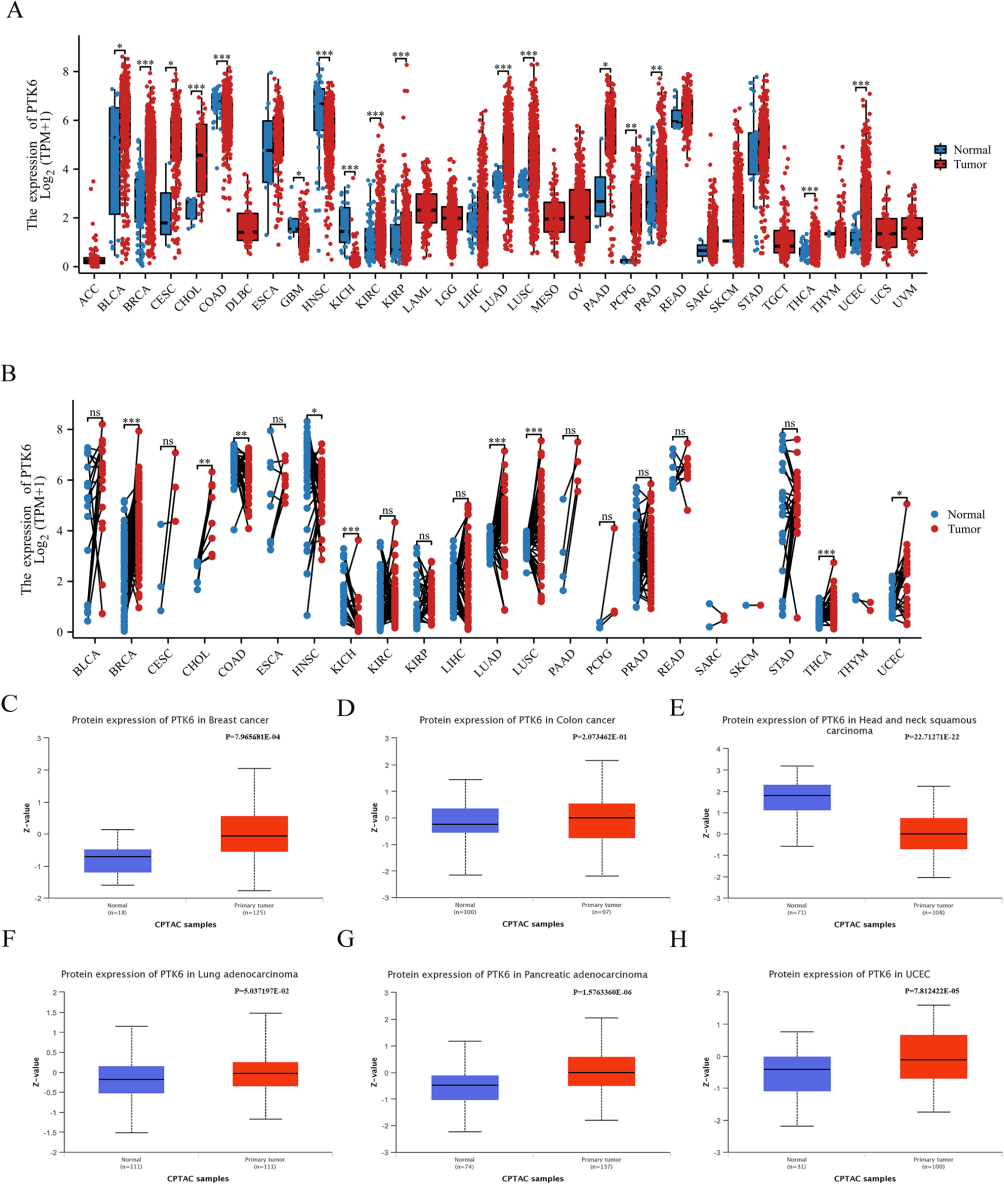
PTK6 expression in pan-cancer analysis. **A** Expression of PTK6 in tumor and normal tissues in TCGA + GTEx dataset. **B** Expression of PTK6 in tumor and paired normal tissues samples. **C**–**H** Protein levels of PTK6 in LUAD and normal tissues samples

PTK6 mRNA expression was discovered to be up-regulated in various tumors, as shown in Fig. 1A, such as CESC, LUAD, BRCA, KIRP, PRAD, UCEC, LUSC, THCA, PAAD, PCPG, BLCA, and CHOL. In addition, the level of PTK6 mRNA was also significantly increased in tumor compared to their adjacent normal tissues (Fig. 1B). In short, PTK6 proves to be up-regulated in pan-cancer.

In addition, the protein levels of PTK6 have been identified to be up-regulated in BRCA, LUAD, PAAD, and UCEC (Fig. 1C–H). Additionally, a PPI network has been established to highlight the relationship between PTK6 and CDK6, HIF1A, EPAS1, KHDRBS1, KHDRBS2, KHDRBS3, STAP2, SFPQ, CBL, ARHGAP35 (Fig. S1).

**Fig. 2:**
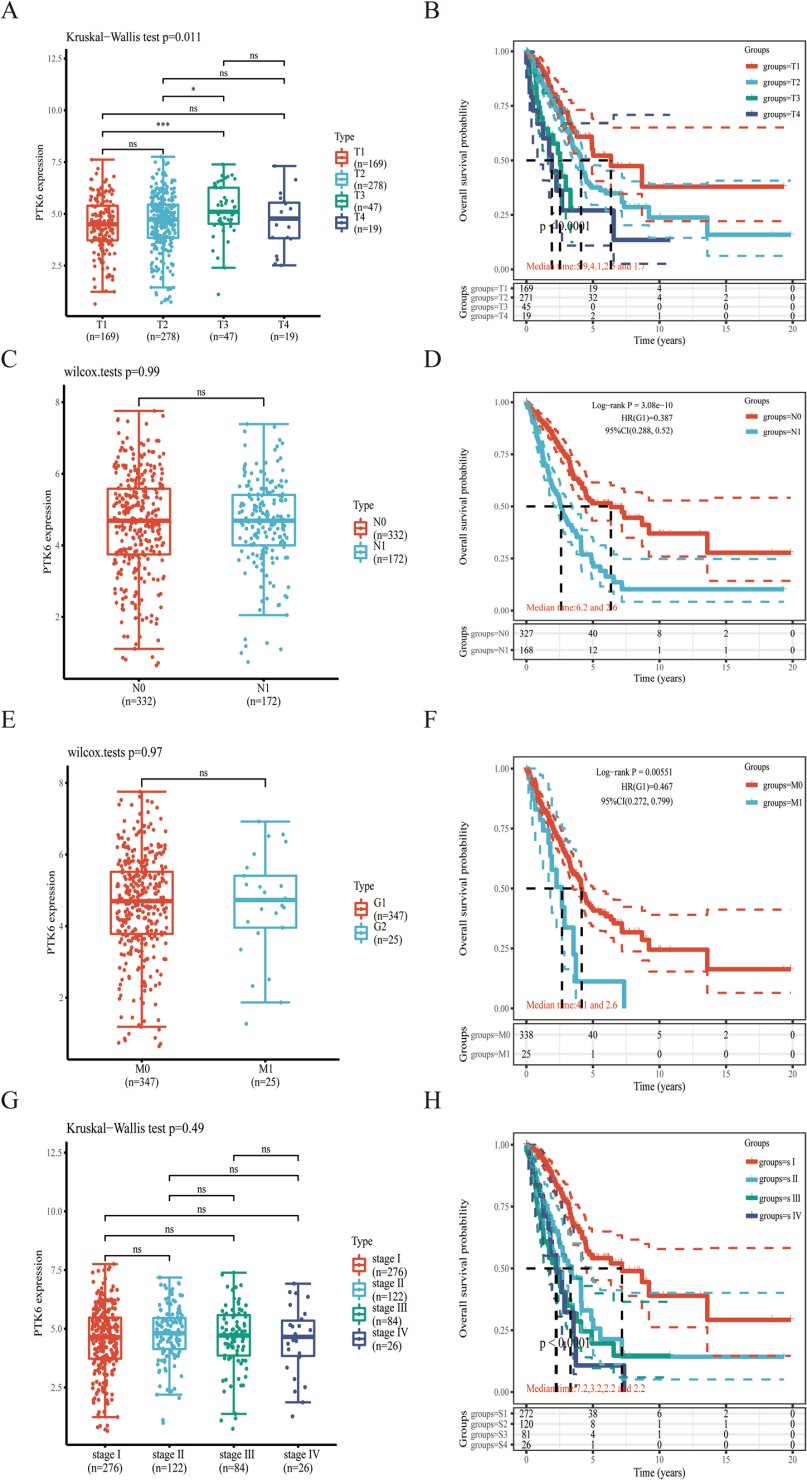
The relationship between the expression of PTK6 and clinicopathological features. **A**, **B** The relationship between PTK6 expression and T stage along with OS. **C**, **D** The relationship between PTK6 expression and N stage along with OS. **E**, **F** The relationship between PTK6 expression and M stage along with OS. **G**, **H** The relationship between PTK6 expression and stage along with OS. *p < 0.05, **p < 0.01, ***p < 0.001, ****p < 0.0001. ns represents no significance

Then, PTK6 expression has been examined at different stages of tumor. PTK6 expression was found to be extensively up-regulated in advanced stages of KIRC, PAAD, SKCM, THCA, ACC, BRCA, ESCA, HNSC, and KICH (Fig. S2). Interesting, we noticed that LUAD patients expressed a higher level of PTK6. Whereas, the baseline of PTK6 in LUAD is summarized in Table 1. The findings suggest a potential association between PTK6 expression levels in LUAD patients across various T, N, M, and stage and their prognostic implications (Fig. 2). Specifically, the results demonstrate a correlation between PTK6 expression and pathologic T stage, with higher levels of PTK6 observed in the T3 group compared to the T1 group, and a corresponding poor overall survival (OS) probability in the T3 group (Fig. 2A, B). There was no difference in N, M stage and stage (Fig. 2C–H). This implies that tumor aggressiveness and size may be related with PTK6 in LUAD.

**Table 1:** Baseline table of clinicopathological features of patients with different PTK6 expression in LUAD

Characteristics	Low expression of PTK6	High expression of PTK6	P value
n	520	521	
Age, n (%)			0.633
≤ 65	220 (21.7%)	228 (22.5%)	
> 65	286 (28.2%)	279 (27.5%)	
Pathologic T stage, n (%)			0.018
T1 and T2	451 (43.4%)	425 (40.9%)	
T3 and T4	67 (6.5%)	95 (9.2%)	
Pathologic N stage, n (%)			0.420
N0	341 (38%)	329 (36.6%)	
N1	109 (12.1%)	119 (13.3%)	
Pathologic M stage, n (%)			0.060
M0	399 (49.3%)	378 (46.7%)	
M1	11 (1.4%)	21 (2.6%)	
Pathologic stage, n (%)			0.329
Stage I and stage II	419 (40.7%)	409 (39.7%)	
Stage III and stage IV	94 (9.1%)	107 (10.4%)	
Smoker, n (%)			0.906
No	47 (4.6%)	48 (4.7%)	
Yes	461 (45.4%)	459 (45.2%)	
OS event, n (%)			0.155
Alive	305 (29.3%)	328 (31.5%)	
Dead	215 (20.7%)	193 (18.5%)	
DSS event, n (%)			0.361
No	365 (38.3%)	379 (39.8%)	
Yes	110 (11.5%)	99 (10.4%)	
PFI event, n (%)			0.291
No	325 (31.2%)	342 (32.9%)	
Yes	195 (18.7%)	179 (17.2%)

### DNA methylation levels with PTK6

**Fig. 3:**
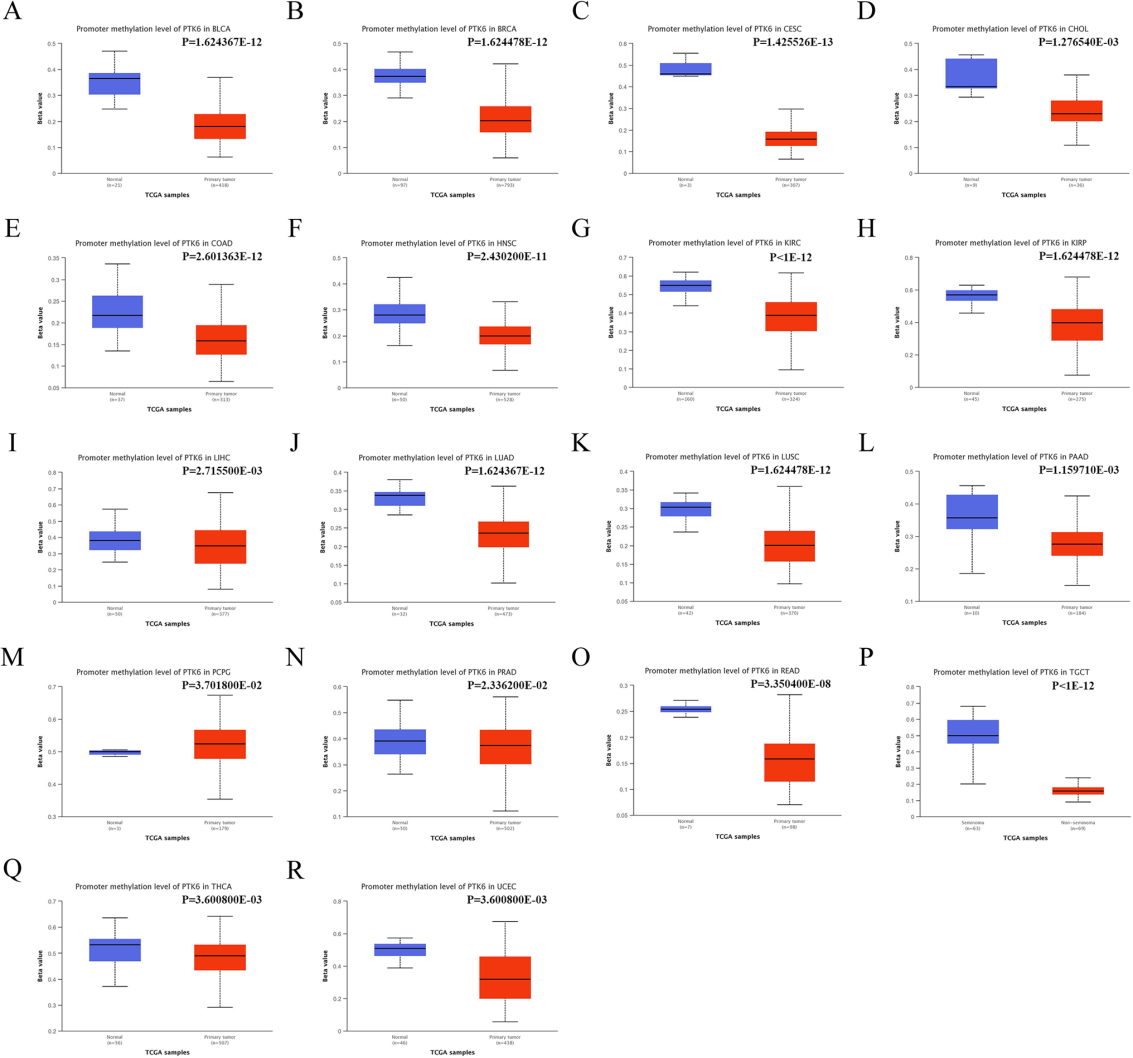
DNA methylation in PTK6. **A**–**R** promoter methylation level of PTK6 in pan-cancer

Therefore, the objective of our study was to assess the promoter methylation status of PTK6 across a range of cancer types. Significant down-regulations of methylation levels were demonstrated in various cancer types, including BLCA, BRCA, CESC, CHOL, COAD, ESCA, KIRC, KIRP, LUAD, LUSC, LIHC, PAAD, PRAD, READ, and SKCM, consistent with mRNA expression levels through spearman correlation analysis (Fig. 3A–R).

### PTK6 expression and patients prognosis

**Fig. 4:**
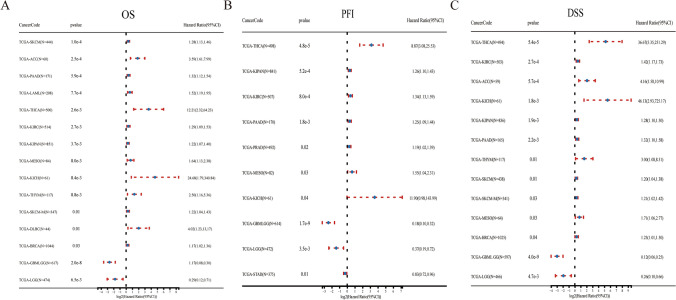
The forest plots were employed to assess the expression of PTK6 and various prognostic markers across diverse cancer types. The statistical analysis revealed a significant association (p < 0.05) between the gene and cancer risk, as evidenced by a Hazard Ratio (95% CI) greater than 1. Conversely, a Hazard Ratio less than 1 indicated a protective effect of the gene. **A** OS; **B** PFI; **C** DSS

Further, Kaplan–Meier analysis was used to estimate potential prognostic value of PTK6. Higher PTK6 expression was associated with poor OS in SKCM, ACC, PAAD, LAML, THCA, KIRC, KIPAN, MESO, KICH, THYM, SKCM, DLBC and BRCA (Fig. 4A) While PTK6 was correlated with poor progression Free Interval (PFI) in THCA, KIPAN, KIRC, PAAD, PRAD, MESO and KICH (Fig. 4B). Likewise, higher PTK6 expression was also linked to poor disease-specific survival (DSS) in THCA, KIRC, ACC, KICH, KIPAN, PAAD, THYM, SKCM, MESO and BRCA (Fig. 4C). These results reveal that PTK6 might function as an independent prognostic marker in pan-cancers.

### Functional enrichment analysis of PTK6

**Fig. 5:**
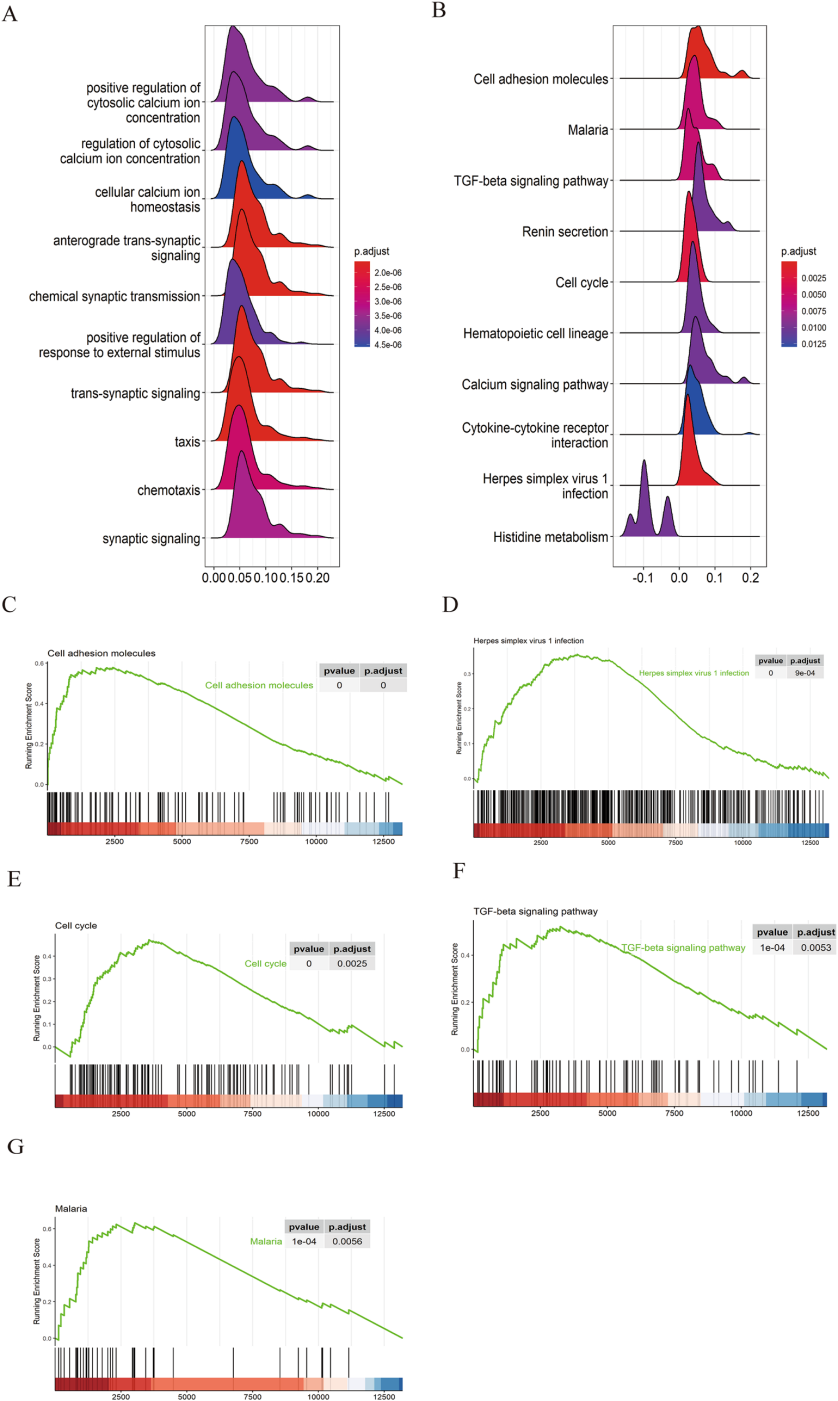
Function enrichment analysis with PTK6 in LUAD. **A** GO analysis with PTK6. **B** KEGG analysis with PTK6. **C**–**G** Gene set enrichment analysis (GSEA) indicating that tumor hallmarks were enriched in PTK6 high group

GSEA, KEGG and GO enrichments was used to identify functional pathways associated with PTK6 in LUAD. GO enrichment results demonstrated that PTK6 overexpression is associated with positive regulation of synaptic signaling, cytosolic calcium ion concentration, and other signaling pathways (Fig. 5A). Indicating that PTK6 could impact multiple pathways, the KEGG results revealed that pathways involving cytokine-cytokine interactions, cell adhesion molecules, TGF-β signaling pathway, cell cycle, and calcium signaling pathway were enriched in PTK6 high expression (Fig. 5B). Furthermore, PTK6-associated pathways were identified with GSEA analysis, the results indicated that pathways related to cell cycle, renin secretion, the TGF-β signaling pathway, and cell adhesion molecules were found to be enriched in high PTK6 expression (Fig. 5C–G).

### Correlation of PTK6 in immune infiltration

In order to deepen our understanding of the relationship between PTK6 and immunity, a comprehensive analysis of 22 immune cell populations was conducted, with the aim of elucidating the intricate interplay between PTK6 and immune cells across various types of cancer (Fig. S3). The results demonstrated a positive correlation between PTK6 and Tregs, as well as a negative correlation with CD8+ and CD4+ T cells across most tumor types. Furthermore, PTK6 was observed to be associated with NK cells, neutrophils, Myeloid dendritic cells, mast cells, and monocytes. Notably, there was a positive correlation with these cell types but a negative correlation with B cells, macrophages M1 and macrophages M2.

**Fig. 6:**
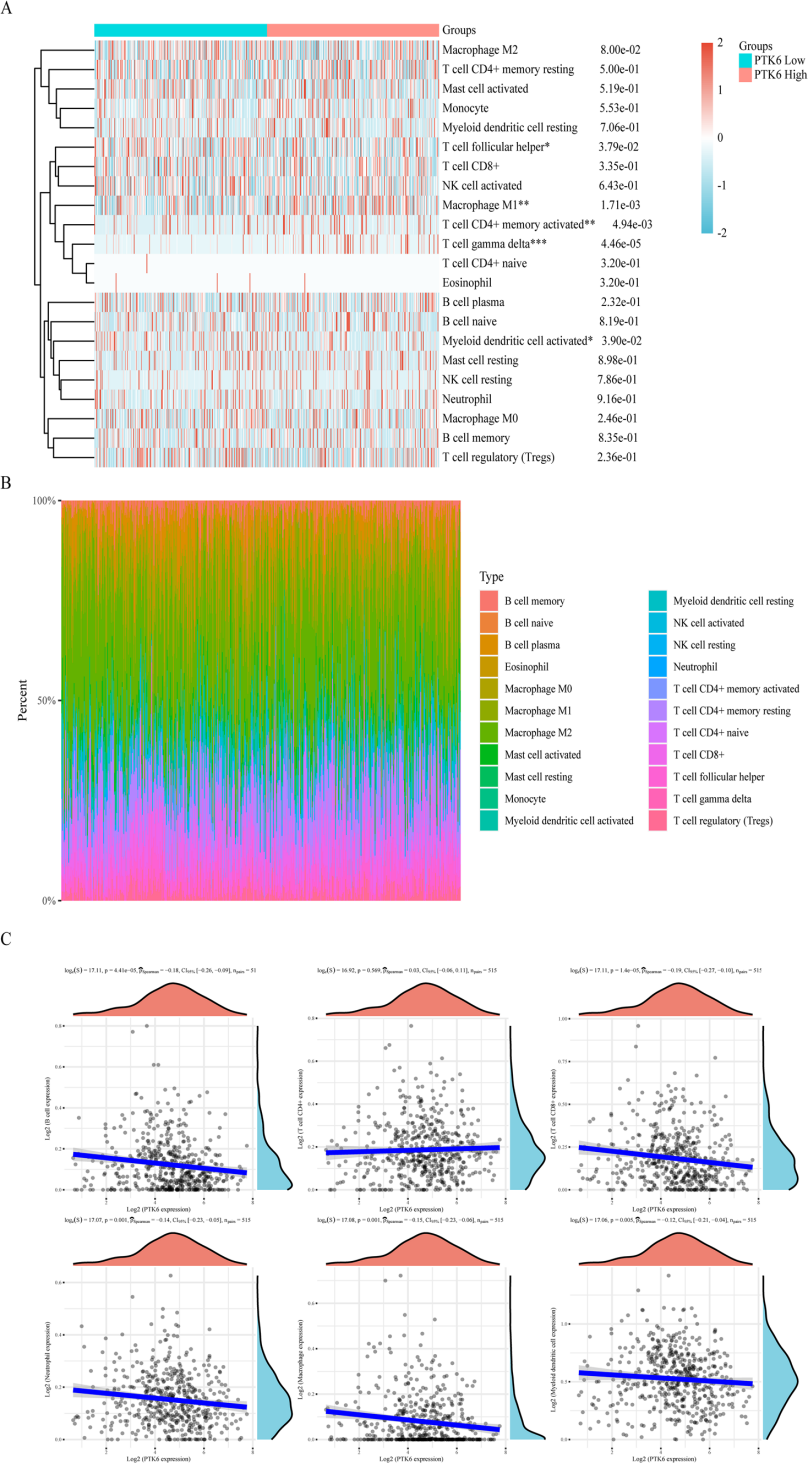
Immune cells with PTK6 in LUAD. **A**, **B** The CIBERSORT algorithm was used to estimate immune cells in patients with LUAD. **C** Correlations of immune cells and PTK6 were evaluated using the spearman method. *p < 0.05, **p < 0.01, ***p < 0.001, ****p < 0.0001. ns represents no significance

As illustrated in Fig. 6, PTK6 was mainly negatively correlated with macrophages M1 and T cells gamma delta. Further investigations were performed to explore the correlation of PTK6 and immune cells. The results demonstrated to be associated with the infiltration of T cell follicular helper, Macrophage M1, T cell CD4+ memory activated, T cell gamma delta, myeloid dendritic cell activated in LUAD (Fig. 6A, B). Then, we evaluated the interactions between PTK6 and different immune cells. As shown in Fig. 6C, PTK6 was negatively correlated with B cell (cor = − 0.18, p < 0.001), CD8+ T cell (cor = − 0.19, p < 0.001), Neutrophil (cor = − 0.14, p = 0.001), Macrophage (cor = − 0.15, p = 0.001) and Myeloid dendritic (cor = − 0.12, p < 0.01). All of those results confirm that PTK6 overexpression is an indicator in tumor immune microenvironment suppression.

### Correlation of PTK6 expression with immunomodulation-related genes and chemokines

**Fig. 7:**
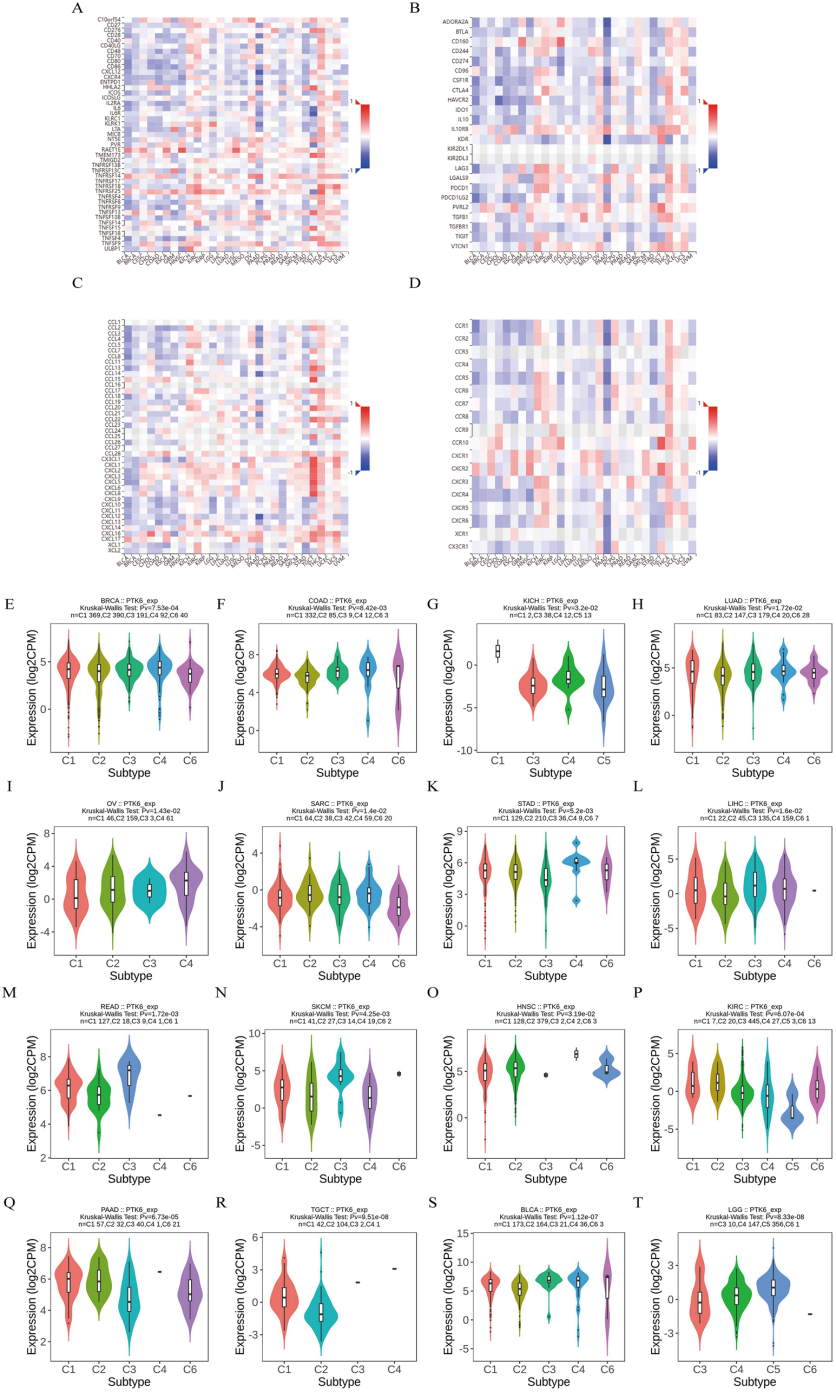
Immune infiltration with PTK6 in pan-cancer. **A**–**D** Correlations of immune modulators and PTK6 in pan-cancer. **E**–**T** Correlations of immune subtypes and PTK6 in pan-cancer

Next, we examined the possible relationships between PTK6 expression and immunomodulators and chemokines. We discovered that PTK6 expression was positively correlated with TNFSF9, TNFSF13, TNFRSF14, TNFRS25, and RAET1E. While negatively correlated with CD27, CD276, CD28, CD40, CD40LG, CD48, CD70, CD80, CD86, and CXCL12 (Fig. 7A). Similarly, the results suggested that PTK6 and RAETE1 had the strongest positive association in HSNC, LUSC, ESCA, and CESC samples, whereas PTK6 and CD276 had the strongest negative correlation in TGCT, THCA, and PRAD samples. In order to regulate the immune response to tumors, exploring immune suppression is essential. According to this study, PTK6 expression was positively correlated with CD160, IL10RB, and PVRL2 while being negatively correlated with ADORA2A, BTLA, CSF1R, and KDR in the other tumor types (Fig. 7B).

Then, investigations on the particular relationship between PTK6 expression and chemokines with their receptors in pan-cancer analysis demonstrated that PTK6 expression was significantly positively attributed to the levels of the chemokines CCL13, CCL15, CX3CL1, CXCL1, CXCL2, CXCL3, CXCL5, CXCL6, and CXCL8 in the majority of malignancies. Furthermore, PTK6 expression was inversely linked with the expression of CCL2, CCL3, CCL4, CCL5, CCL8, and CXCL12 (Fig. 7C, D).

Afterwards, the correlation between different immune subtypes and PTK6 expression in pan-cancer were examined. The immune subtypes were categorized into six distinct types: C1 (wound healing), C2 (IFN-gamma dominant), C3 (inflammatory), C4 (lymphocyte depleted), C5 (immunologically quiet), and C6 (TGF-b dominant) [32]*.* It is noteworthy that PTK6 expression is most pronounced in the C4 subtype, including *BRCA*, *COAD*, *KICH*, *LUAD*, *OV*, *SARC*, *SARC STAD* (Fig. 7E–T). The C4 subtype is characterized by a heightened macrophage profile, concurrent Th1 cell suppression, and an elevated M2 cell response and of these subtypes, the C3 subtype demonstrates the most favorable prognosis, while the C2 and C1 subtypes exhibit a poorer prognosis despite a significant immune component [32]. Conversely, the C4 and C6 subtypes display the lowest overall survival rates. All this could be attributed to T-cell dysfunction triggered by PTK6.

### Association between PTK6 expression and ICB response

**Fig. 8:**
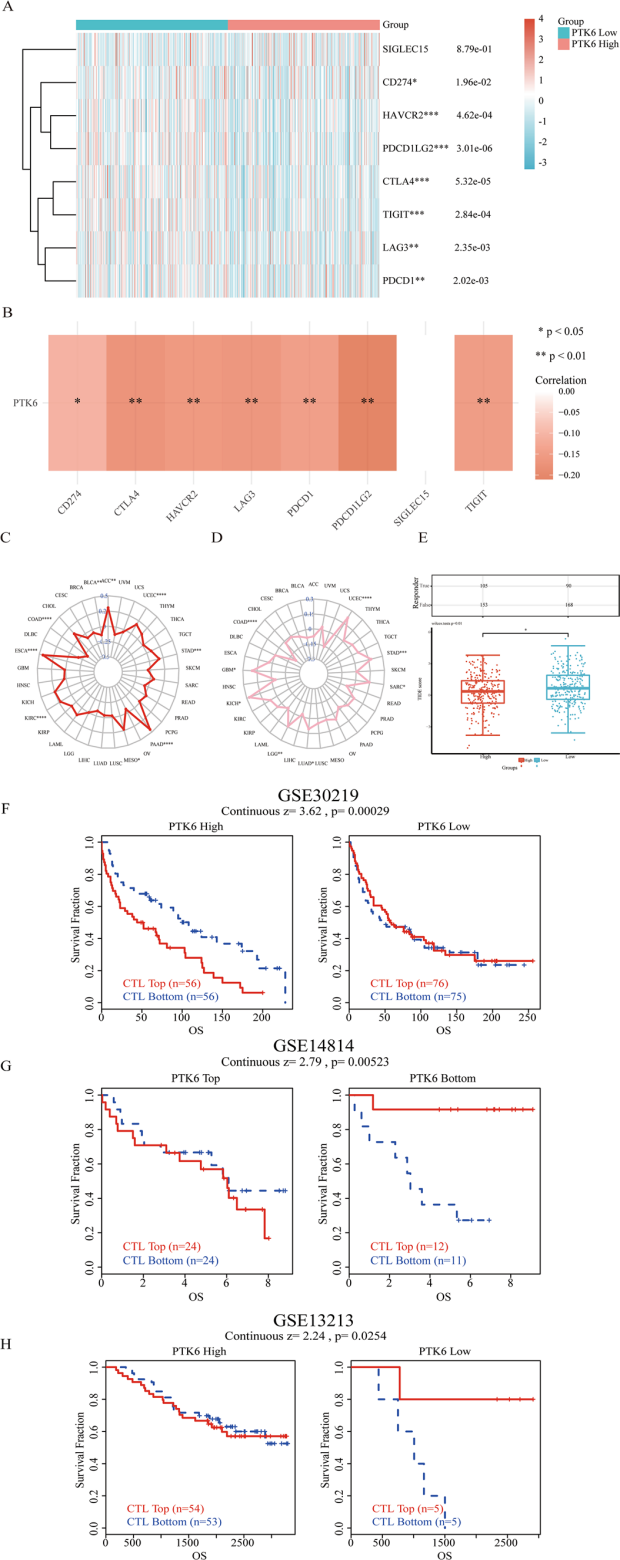
Immune therapy in PTK6. **A**, **B** Correlation of PTK6 expression and immune checkpoints (SIGLEC15, CD274, HAVCR2, DPCD1LG2, CTLA4, TIGIT, LAG3, PDCD1). **C**, **D** Correlations of PBK expression with TMB and MSI in pan-cancer. **E** TIDE scores of different PTK6 expression subgroups. **F**–**H** Prognosis of different datasets of immune therapy cohorts, including GSE30219 (**F**), GSE14814 (**G**), GSE13213 (**H**)

Immunotherapy needs to concentrate on immune checkpoints. The relationship between the expression of PTK6 and eight immune checkpoint genes were further determined by our investigations in LUAD. It’s interesting to take into account that PTK6 obviously associated with the levels of CD274, HAVCR2, PDCD1LG2, CTLA4, TIGIT, LAG3, PDCD1 expression (Fig. 8A). Thus, in order to figure out the connections between PTK6 and certain checkpoints in detail, we analyzed the association between PTK6 and these eight checkpoints. PTK6 was shown to be firmly adversely correlated with all immune checkpoints except SIGLEC15 (Fig. 8B).

Further, tumor mutational burden (TMB) and microsatellite instability (MSI) are considered to be important factors reflecting prognosis and immunotherapy response [33]. It has been reported that high TMB levels are associated with high MSI levels. Thus, the relationship between TMB, MSI and PTK6 expression may provide guidance for clinical immunotherapy of tumors. As a result, the positive correlation between PTK6 expression and high TMB was significant in PAAD, ESCA, KIPAN, ACC, KICH, STES, UCEC and STAD (Fig. 8C). Similarly, the positive association between PTK6 expression and MSI was significant in GBM, KICH, UCEC, SARC, GBM, LGG, TGCT, STAD, STES, THCA, LUAD, and KIRC (Fig. 8D).

In addition, to further predict PTK6’s potential as an immunotherapy target, we evaluated TIDE scores of PTK6 in different expression groups (Fig. 8E).

Notably, the low expression group of PTK6 exhibited a reduced number of responders, while the high expression group demonstrated an increased number of responders. Furthermore, the PTK6 low-expression group displayed significantly higher Tide scores compared to the high-expression group. Conversely, elevated TIDE scores were associated with diminished efficacy of ICB and shorter survival post ICB administration [24]. This further substantiates the potential of PTK6 as a promising target for immunotherapy. The GEO cohort (GSE30219) of Lung patients were analyzed using the TIDE database, comparing with the PTK6 low expression group, the PTK6 high expression group had a poor OS (Fig. 8F) [34]. While, the CTL high group had a worse survival outcome than the CTL low group in the PTK6 high expression group. Both GSE14814 and GSE13213@PRECOG had the same outcomes as previous cohort [35, 36] (Fig. 8G, H). This may cause by T cell dysfunction rather than T cell amounts.

### Tumor proliferation can be inhibited by PTK6 suppression

**Fig. 9:**
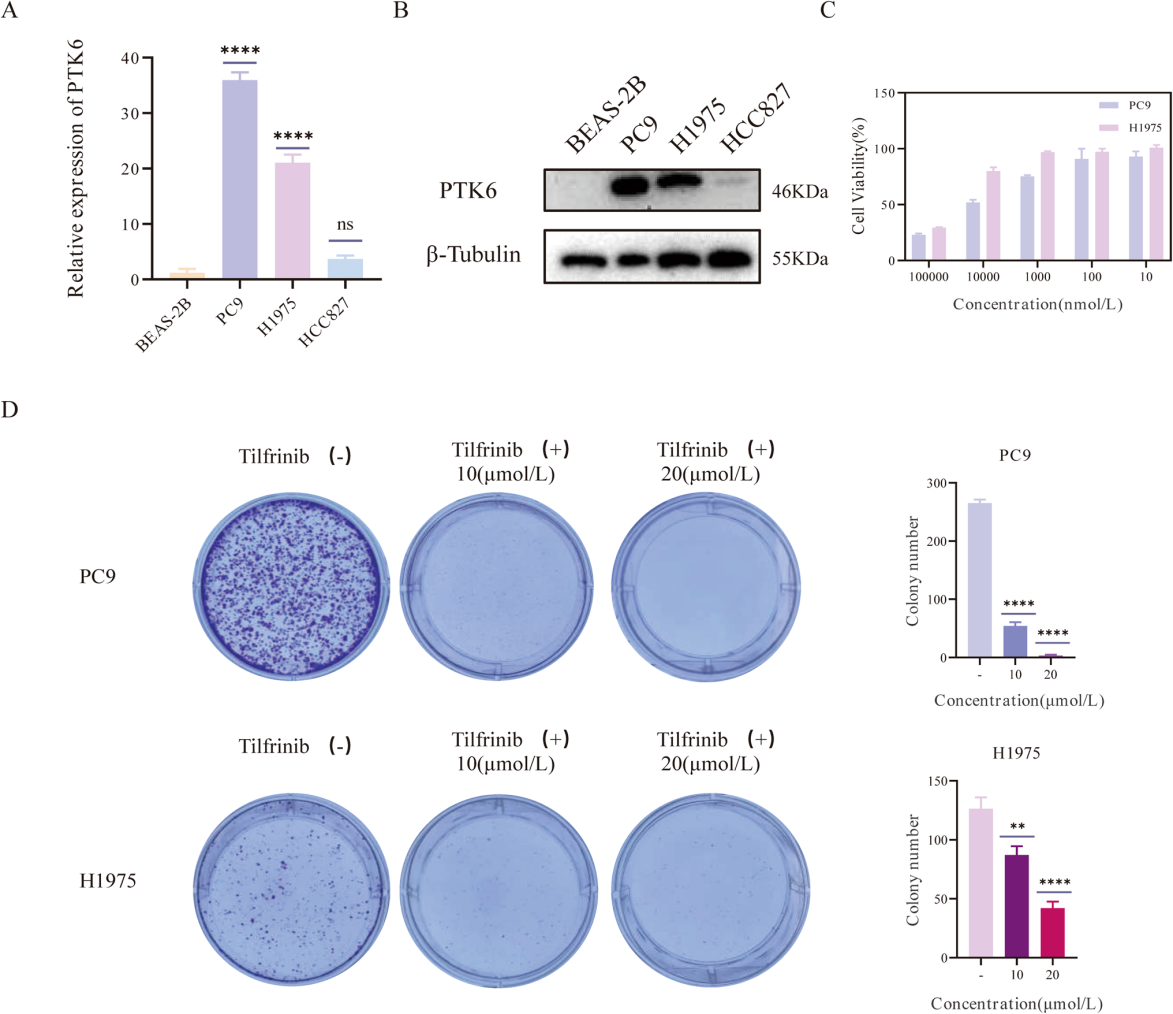
Expression levels of PTK6 in BEAS-2B, PC9, H1975, A549 and the role of Tilfrinib as a PTK6 inhibitor in lung cancer cell lines. **A** PTK6 expression level in lung cancer cell lines was analyzed through RT-qPCR. **B** Western blotting of PTK6 expression in lung cancer cell lines. **C** Cell viability of H1975, PC9 cells was detected by CCK-8 after different concentrations of Tilfrinib. **D** Anti-colony formation of Tilfrinib on PC9 and H1975. *p < 0.05, **p < 0.01, ***p < 0.001, ****p < 0.0001. ns represents no significance

Tilfrinib is a highly effective and selective PTK6 inhibitor, showing good anti-proliferative activity in tumors [37]. To further investigate the role of PTK6 in the evolution of LUAD, first we utilized qPCR to evaluate PTK6 expression in LUAD (Fig. 9). We employed four different cell lines including BEAS-2B, PC-9, H1975, and HCC827. Comparing to BEAS-2B, PTK6 was found to be evidently expressed in PC9 and NCI-1975 (Fig. 9A). In addition, the same results were also identified using western blot (Fig. 9B). Following that, PC9 and H1975 were employed for next experiments. Next, we conducted the CCK-8 assay to determine Tilfrinib’s anti-proliferative effect in LUAD. As the Fig. 9C illustrates, cell viability declined as the concentration of tilfrinib increased, with 10um being the lowest. Furthermore, colony formation assays were performed to confirm the proliferation of cells following PTK6 inhibition (Fig. 9D). The three concentrations of Tilfrinib we used were 0 μM, 10 μM, and 20 μM. The results indicated that cell proliferation was suppressed as Tilfrinib concentration increased. The findings indicated that PTK6 exhibits high levels of expression in PC9 and H1975 cell lines, and significantly, tilfrinib demonstrates efficacy in suppressing this expression. Furthermore, following the inhibition of PTK6 overexpression, a reduction in cell viability and proliferation was observed in these lung cancer cell lines.

### Tumor invasion can be inhibited by PTK6 suppression

**Fig. 10:**
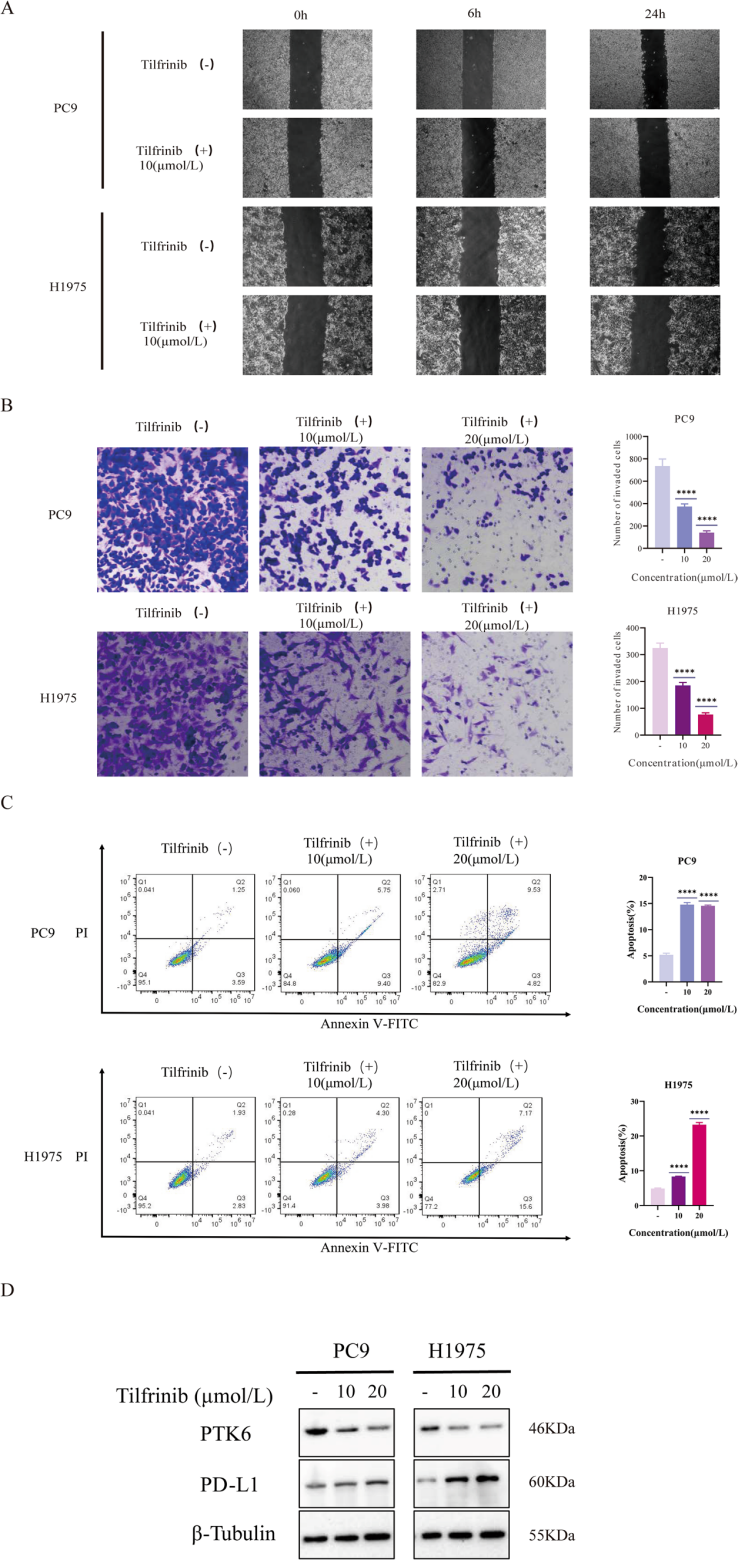
Tilfrinib inhibits the growth in lung cancer cell lines and regulates the expression level of PD-L1. **A** The wound healing assay was performed to determine the wound healing migration activity after different concentrations of Tilfrinib. **B** Invasion ability was determined using the Transwell invasion assay after different concentrations of Tilfrinib. **C** Tilfrinib induced cell apoptosis in PTK6-positive lung cancer cells. **D** Inhibition of PTK6 with Tilfrinib leads to up-regulation of the expression of PD-L1. ****p < 0.0001

To investigate whether inhibition of PTK6 can prevent tumor cell invasion, we conducted wound healing and invasion experiments. Two concentrations of 0 μm and 10 μm were used. We record results based on 0 h, 6 h and 24. The wound healing results demonstrated that the scratch area of Tilfrinib (+) group was larger than that of Tilfrinib (−) group with the time slid (Fig. 10A). While, in the invasion experiment, we set three concentrations of 0 μm, 10 μm and 20 μm of Tilfrinib. The results indicated that the invasion rate decreases with the increase of the concentration. These results revealed that inhibition of PTK6 can reduce the invasion of LUAD (Fig. 10B).

### PTK6 suppression inhibited tumor apoptosis and up-regulated PD-L1

Finally, Annexin V/PI staining experiments were performed to examine whether suppressing PTK6 could diminish tumor cell apoptosis (Fig. 10C). We employ three different concentrations: 0 μm, 10 μm, and 20 μm. The results displayed that when PTK6 was suppressed, apoptosis increased. After that, we examined PD-L1 expression. The results indicated that PTK6 was substantially suppressed in all three groups as concentration increased. Furthermore, there was a obvious increasing of PD-L1 expression (Fig. 10D).

## Discussion

PTK6 is a tyrosine kinase belonging to the PTK6 family, which also includes FRK and SRMS [38]. PTK6 may collaborate with a range of growth factor receptors, including as the ERBB family, IGF1R, and MET, as well as a variety of signaling pathways, such as hypoxia, p27/CDK/Cyclins, ERK1/2/P38 to promote tumor progression [39–41].

PTK6 is now the focus of research in prostate, colorectal, and particularly breast cancer, which is frequently highly expressed in tumor and associated with poor prognosis [12, 13, 42]. PTK6 expression levels in breast cancer are correlated with a higher probability of tumor cell invasion, migration, and metastasis [15, 16, 39, 43, 44]. Besides, PTK6 also stops breast cancer cells from going into autophagy, ensuring their survival [45]. In prostate cancer, increased PTK6 expression is associated with poor patient prognosis and recurrence [42]. Initially, bioinformatics analysis was employed to investigate the association between PTK6 expression and tumor. The findings of our study indicate that PTK6 mRNA and protein levels exhibit significant upregulation across various cancer types. As for methylation analysis, historically, cancer cell initiation has been predominantly ascribed to genetic mutations, positing them as the fundamental catalysts for cancer. Nevertheless, recent research has shed light on the significant role of epigenetics in gene regulation and cellular mechanisms [46]. Notably, the disruption of epigenetic processes can contribute to tumor formation. Epigenetic alterations, including DNA methylation and histone acetylation, are reversible phenomena that can be targeted for therapeutic intervention [47]. Further methylation analysis demonstrates a decrease in PTK6 promoter methylation levels in pan-cancer, aligning with our previous expression studies. Additionally, PTK6 overexpression displays a positive correlation with T stage and a negative correlation with prognosis. Subsequent survival analysis reveals PTK6 to be a risk factor for OS, PFI, and DSS in multiple tumor types. Furthermore, our in vitro experiments have substantiated that the inhibition of PTK6 effectively enhances the proliferation, invasion, migration, and apoptosis of LUAD cells. Consequently, the overexpression of PTK6 presents a promising avenue for clinical biological therapy.

In order to further investigate the potential oncogenic mechanism of PTK6 in LUAD, examinations were conducted to explore the relationship between PTK6 and the tumor immune microenvironment. Initially, a correlation analysis was performed to assess the association between PTK6 and immune cells. The results revealed a significant correlation between PTK6 and T cell follicular helper, Macrophage M1, T cell CD4+ memory activated, T cell gamma delta, and myeloid dendritic cell activated. Subsequent analysis demonstrated a negative correlation between PTK6 and the majority of immune cells, suggesting a potential link between PTK6 overexpression and immunosuppression.

Furthermore, we investigated the association between PTK6 and various immunomodulators. The findings revealed a significant inverse relationship between PTK6 and immuno-stimulators. Whereas, immune-subtype analysis indicated that PTK6 exhibited the highest expression in the C4 subtype. To ascertain the potential of PTK6 as a target for immunotherapy, we also examined the correlation between immunotherapy and PTK6. Remarkably, our results demonstrated a negative correlation between PTK6 and nearly all immune checkpoints, providing further evidence that overexpression of PTK6 leads to immune suppression. Moreover, PTK6 exhibited a significant correlation with TMB and MSI across various tumor types. Specifically, in the case of LUAD, PTK6 displayed a positive correlation with MSI, and elevated MSI levels were indicative of the effectiveness of immunotherapy. Consequently, targeting PTK6 may enhance the immunotherapeutic response, thereby improving the overall survival of LUAD patients. Our experimental findings also demonstrated an increase in PD-L1 expression following PTK6 inhibition, suggesting that a combination of PTK6 inhibitors and PD-L1 inhibitors may yield promising results.

At this point, we conducted research on immunotherapy in three different datasets. The OS was low in the group with high CTL expression and PTK6 expression. This demonstrates that CTL were high in patients with high PTK6 expression, but the prognosis of patients was still bad due to T-cell dysfunction, not because of the low T-cell infiltration. Furthermore, we discovered that PD-L1 expression increased after PTK6 knockdown in lung adenocarcinoma cell lines, and the cohort survival analysis with immunotherapy suggested that T-cell number in the immune microenvironment does not determine the effect of immunotherapy, but rather T-cell function does.

In general, it is commonly believed that a high abundance of T cells is indicative of an effective anti-tumor response and a favorable prognosis [48]. However, our findings demonstrate that the group exhibiting high PTK6 expression possesses a substantial number of T cells, yet patients within this cohort still experience poor survival outcomes. This discrepancy may be attributed to the dysfunctionality of T cells, resulting in an increased presence of immune cells that are unable to effectively combat tumors.

These correlations reveal a potential mechanism by which PTK6 regulates T cell function in LUAD. In addition, we observed a significant association between LUAD and Macrophage M1, myeloid dendritic cell activated, suggesting that PTK6 may inhibit the immune microenvironment in LUAD. Therefore, PTK6 has prognostic value in LUAD. Inevitably, the study has some limitations. Firstly, it is important to note that our analysis is limited to a small number of TCGA and GEO cohorts, thus necessitating the validation of our findings with a larger sample size. Secondly, it is crucial to acknowledge that algorithmic analysis based solely on RNA sequences may not possess sufficient accuracy. Consequently, further experimentation utilizing in vivo experiments are required to investigate the underlying biological mechanisms of PTK6, as well as the interactions between tumor immunity and PTK6 in LUAD. In summary, our study has demonstrated the prognostic significance of PTK6 and its impact on the immune status of LUAD. Specifically, elevated expression of PTK6 in LUAD is associated with reduced levels of immune infiltration and checkpoint expression, thereby potentially influencing the tumor microenvironment.

## Conclusion

This study employs bioinformatics and experimental methods to conduct a comprehensive investigation into the involvement of PTK6 in cancer. The findings indicate a significant overexpression of PTK6 in various tumor types, which correlates with clinicopathological characteristics and unfavorable prognosis. Furthermore, the results demonstrate the association of PTK6 with cell proliferation, invasion, and apoptosis specifically in LUAD. Additionally, PTK6 exhibits a crucial association with the immune microenvironment, and inhibition of PTK6 leads to up-regulation of PD-L1 expression, In conclusion, PTK6 may be a novel potential biomarker for LUAD, and may be synergistic with immunotherapy, thereby enhancing therapeutic efficacy and inhibiting tumor progression.

### Supplementary Information

Below is the link to the electronic supplementary material.

Supplementary file 1 (DOCX 1854 KB)

Supplementary file 2 (PDF 93 KB)

## Data Availability

The data analyzed during the current study are available in TCGA and GEO database with the accession numbers TCGALUAD and GSE30129, GSE14814, GSE13213. The original contributions presented in the study are included in the article; further inquiries can be directed to the corresponding author.
