# Supplementary material for: Identification of immune-associated biomarker for predicting lung adenocarcinoma: bioinformatics analysis and experiment verification of PTK6
Source: Discov Oncol. 2024 Apr 4;15:102. doi: 10.1007/s12672-024-00939-9 (PMC10994900; doi:10.1007/s12672-024-00939-9)
Supplement: Supplementary file 1 — (DOCX 1854 KB) [file 12672_2024_939_MOESM1_ESM.docx]

**
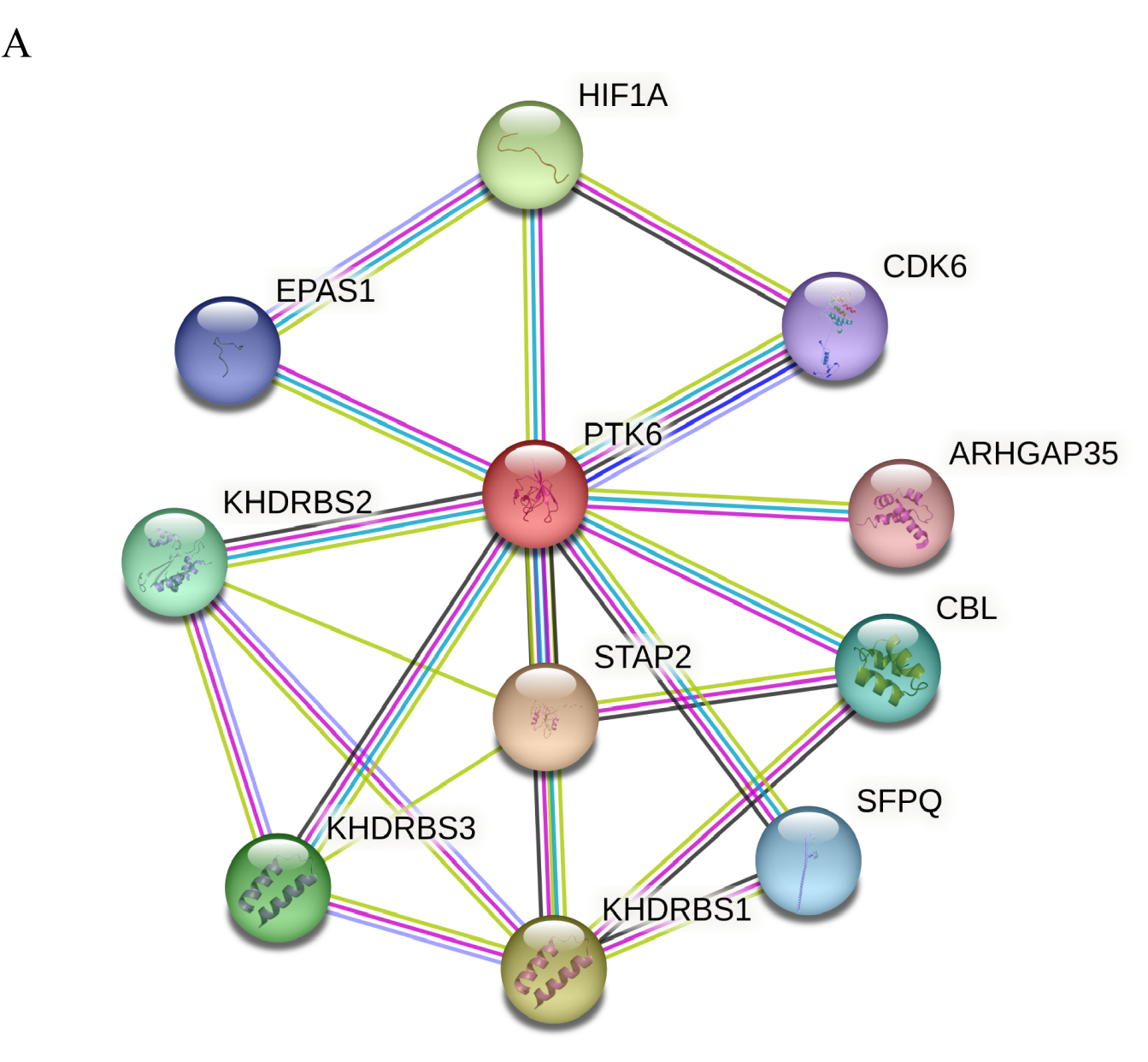
**

Fig. S1 A PPI network has been established to highlight the relationship between PTK6 and CDK6, HIF1A, EPAS1, KHDRBS1, KHDRBS2, KHDRBS3, STAP2, SFPQ, CBL, ARHGAP35.

**
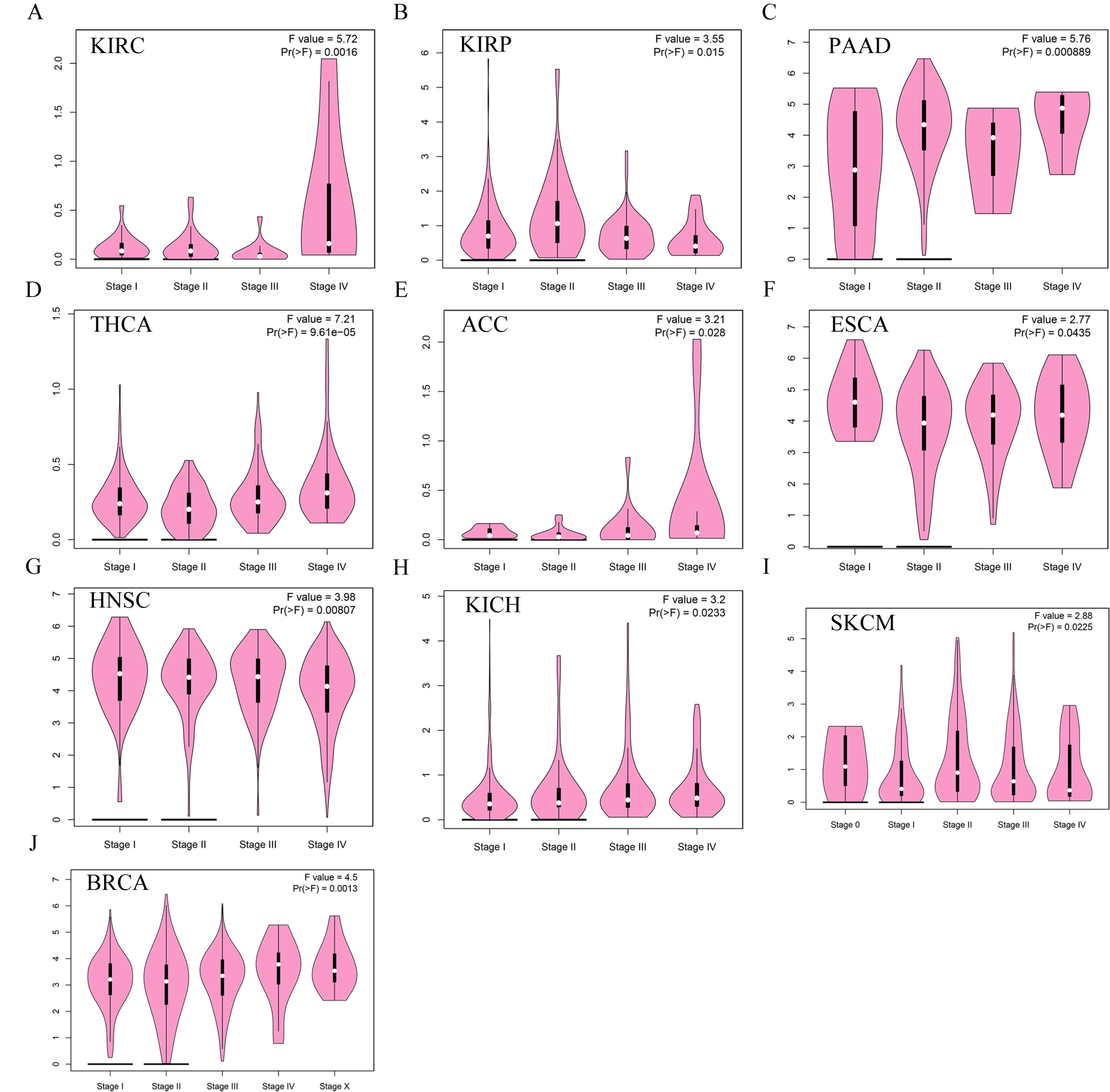
**

Fig. S2 Stage-dependent expression levels of PTK6 (A–J). Main pathological stages of tumors were assessed and compared using TCGA data. The log2 (TPM + 1) for log-scale was used.

**
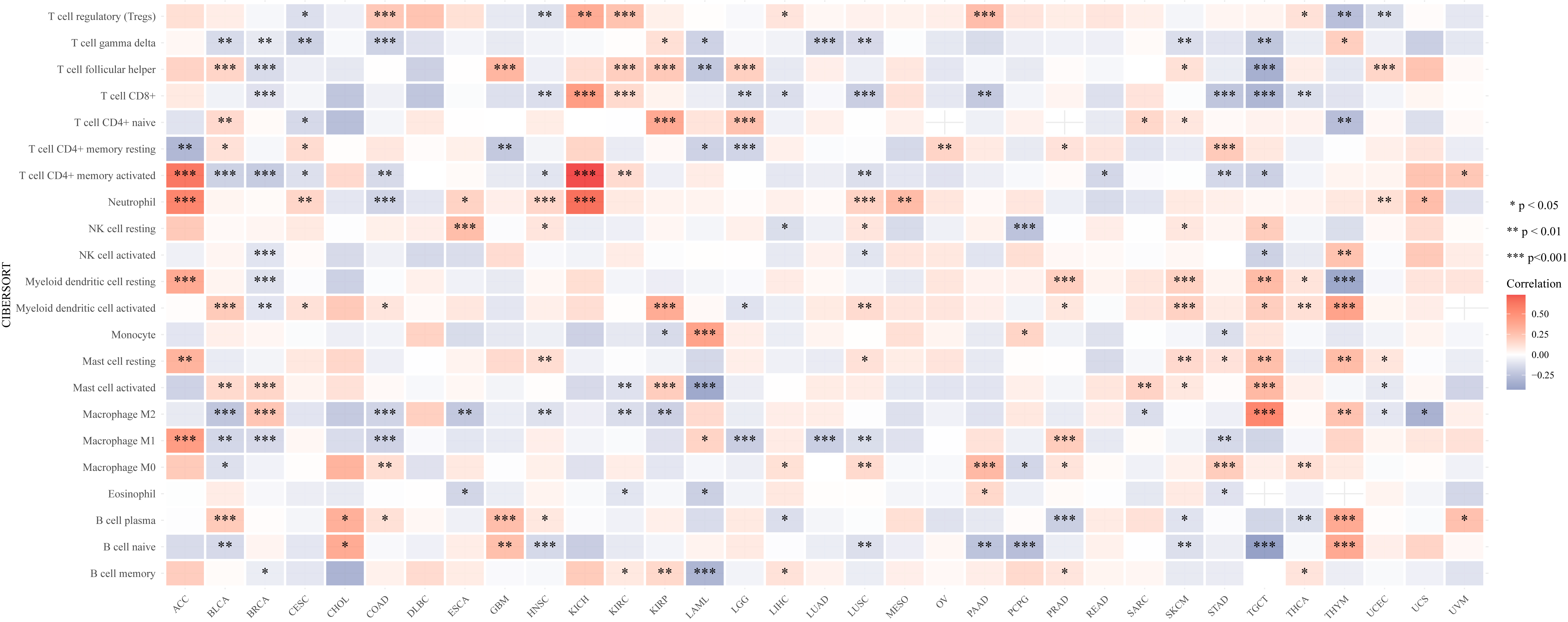
**

Fig. S3 Association of PTK6 with immune cells in pan-cancer, * *p* < 0.05, ** *p* < 0.01, *** *p* < 0.001.
