# Supplementary figures and images for: Identification of immune-associated biomarker for predicting lung adenocarcinoma: bioinformatics analysis and experiment verification of PTK6
Source: Discov Oncol. 2024 Apr 4;15:102. doi: 10.1007/s12672-024-00939-9 (PMC10994900; doi:10.1007/s12672-024-00939-9)

A

PTK6  
46kDa

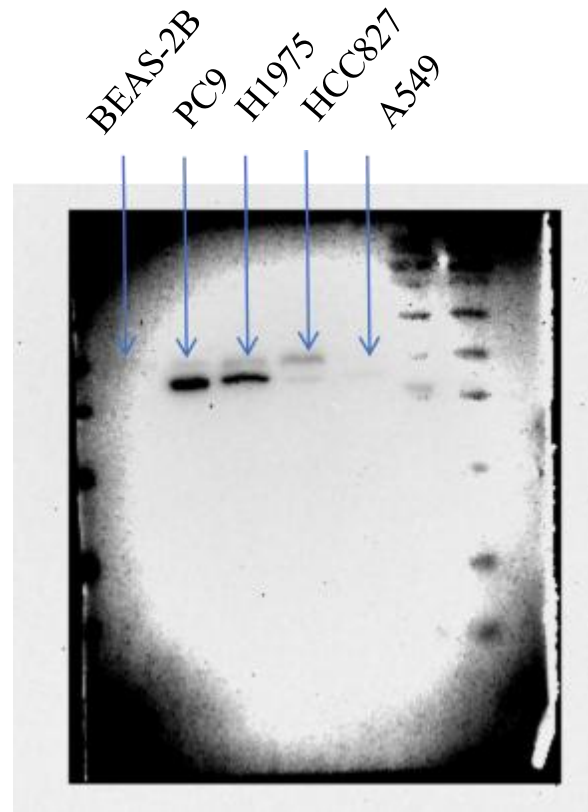

B

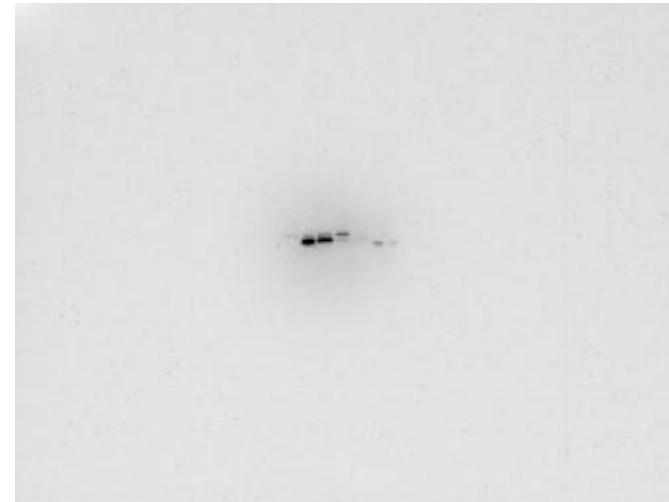

C

$\beta$ -Tubulin  
55kDa

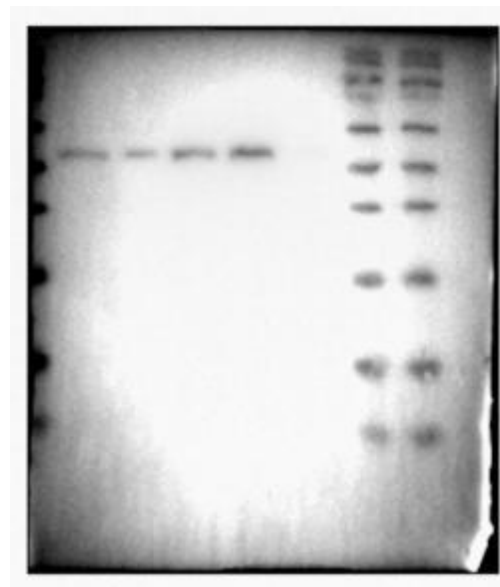

D

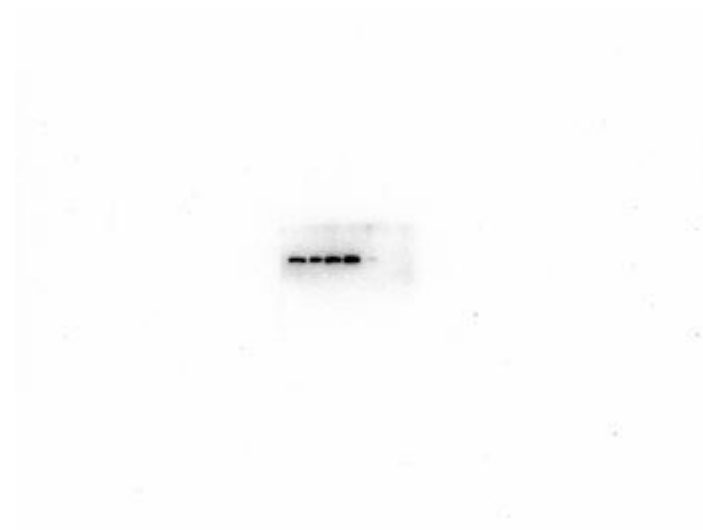

A

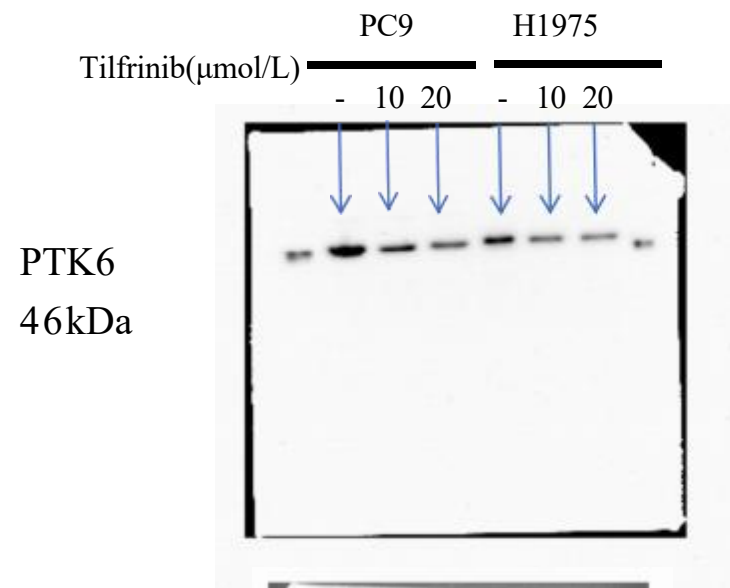

B

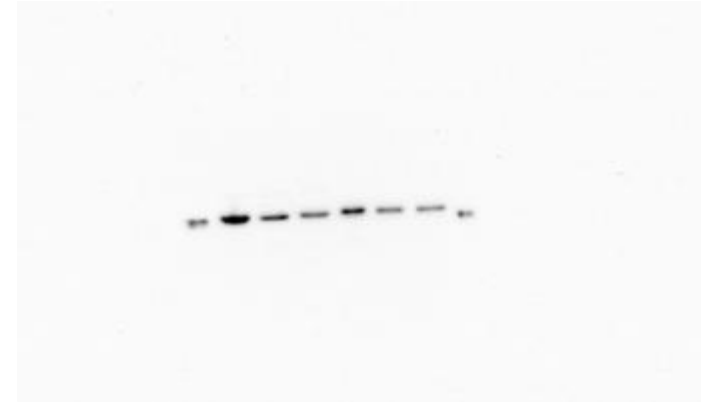

C

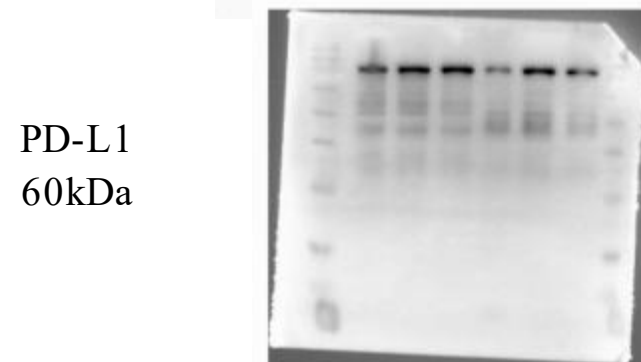

D

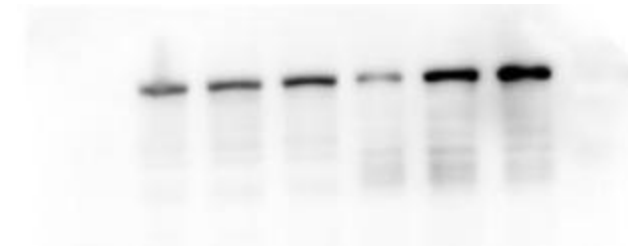

E

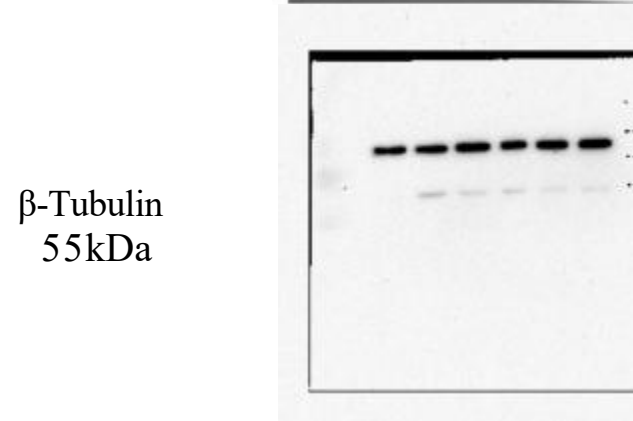

F

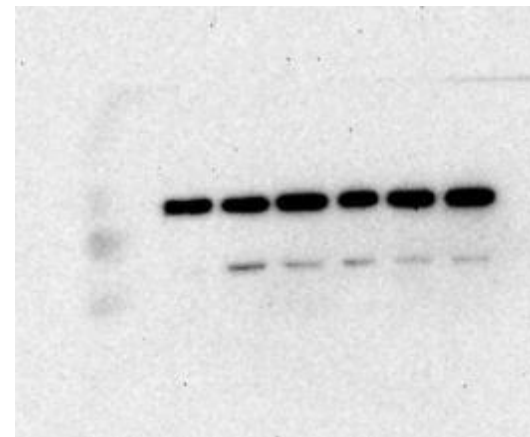

Supplement: Supplementary file 2 — (PDF 93 KB) [file 12672_2024_939_MOESM2_ESM.pdf]
